# Ambient temperature as a proxy indicator for carbon monoxide poisoning risk: deriving exploratory reference points for public health monitoring in Chengdu, China

**DOI:** 10.3389/fpubh.2026.1858128

**Published:** 2026-07-15

**Authors:** Nan Du, Dan Kuang, Xufang Gao, Jingwen Sun, Li Luo, Xiaochen Wang, Cheng Wang, Rong Lu

**Affiliations:** Chengdu Center for Disease Control and Prevention (Chengdu Institute of Health Supervision), Chengdu, Sichuan, China

**Keywords:** carbon monoxide poisoning, Chengdu, distributed lag nonlinear model, health risk communication, risk reference point, temperature, time-stratified case-crossover design

## Abstract

**Objective:**

Low ambient temperature is associated with increased carbon monoxide (CO) poisoning incidents, likely through behavioral pathways such as heating device use and reduced ventilation. This study examines the association between daily mean temperature and CO poisoning emergency medical service (EMS) responses in Chengdu, explores potential population susceptibility, estimates the attributable burden, and proposes exploratory temperature reference points for public health monitoring.

**Methods:**

CO poisoning EMS records from Chengdu (January 1, 2013–December 31, 2025; 4,352 cases across 1,894 days) were matched with daily meteorological and air pollutant data. A time-stratified case-crossover design combined with distributed lag nonlinear models (DLNMs; max lag = 5 days) was employed. Models adjusted for relative humidity, PM_2_.5, wind speed, precipitation, day of week, and holidays, using median temperature (10.5 °C) as reference. Stratified analyses covered age, sex, season, and humidity. Attributable fractions were estimated via Monte Carlo simulations.

**Results:**

A reverse “J”-shaped association was observed, with a cumulative lag 0–5 days minimum morbidity temperature (MMT) of 14.9 °C. Extreme cold (2.9 °C, 2.5th percentile) vs. reference was associated with a cumulative RR of 5.12 (95% CI: 1.74–15.09), peaking at lag 0 (RR = 1.79, 95% CI: 1.47–2.17). Subgroup analyses suggested potential vulnerability among adults aged 20–39 and ≥60 years. Wind speed stratification indicated a 69% higher extreme cold risk on low-wind days (RR ratio = 1.69), though formal interaction testing was non-significant (*p* = 0.94). Non-optimal temperatures were statistically coincident with an estimated 32.9% of EMS responses. Nominal exploratory reference points of 7.3 °C and 6.0 °C were derived; bootstrap validation (1,000 resamples) yielded median estimates of 7.0 °C (95% CI: 6.0–8.0 °C) and 6.0 °C (95% CI: 4.3–6.7 °C).

**Conclusion:**

Low ambient temperature serves as a measurable proxy indicator for conditions associated with elevated CO poisoning risk in Chengdu. The proposed exploratory temperature reference points (7.3 °C and 6.0 °C) may inform targeted safety messaging during winter, though prospective validation is required before operational implementation in warning systems.

## 1 Introduction

Carbon monoxide (CO) poisoning is a preventable public health concern worldwide. CO exposure is associated with the formation of carboxyhemoglobin, which can cause tissue hypoxia and potentially death or delayed neurological sequelae. Globally, an estimated 137 cases and 4.6 deaths per million occur annually (1), with approximately 970,000 poisoning incidents and 41,000 deaths worldwide despite declining age-standardized incidence rates (2). In China, tens of thousands of CO poisoning cases are reported annually, with the highest incidence during winter heating seasons (3).

Emergency medical service (EMS) data provide a unique opportunity to capture the acute health effects of temperature exposure (4). A national study conducted across six Chinese provinces (2013–2019) further demonstrated that each 1 °C rise in daily mean temperature was associated with a 0.50% increase in injury mortality risk, with intentional injuries exhibiting greater sensitivity than unintentional injuries (5). Previous studies have identified low ambient temperature as a key environmental indicator associated with increased CO poisoning risk, likely operating through behavioral adaptations rather than direct physiological pathways. Studies in Jinan, Guangdong, and 31 Chinese cities consistently reported elevated CO poisoning risks during cold periods, with stronger effects in southern regions and lag structures persisting up to 7 days (6–8). Shanghai surveillance data (2007–2018) further identified winter as the peak season, with a rising trend in summer cases (9).

The Chengdu Plain, located in the Sichuan Basin, is characterized by high winter humidity, low wind speed, and poor atmospheric diffusion (10). A recent review of public health vulnerabilities across different regions of China highlighted the need for region-specific studies to inform local public health interventions, including in the Southwest region (11). These unique climatic conditions could influence the temperature-CO poisoning relationship, yet systematic studies in this region are lacking. Recent environmental health studies in Chengdu have demonstrated the importance of considering temperature effects in local populations, further highlighting the need for region-specific assessments (12). Gansu province research indicated that extreme cold disproportionately affects women, adults aged ≥65 years, and CO poisoning hospitalizations (13). Sichuan-specific mortality data showed that CO poisoning predominantly occurs in winter, with adults aged 18–60 years accounting for 80.2% of deaths, primarily due to improper use of gas water heaters (14).

The distributed lag nonlinear model (DLNM) is the standard method for simultaneously assessing nonlinear exposure-response relationships and lag structures (15, 16). DLNM has been widely applied across 36 countries to analyze the delayed and cumulative effects of environmental exposures on diverse health outcomes (17). The time-stratified case-crossover design effectively controls for time-invariant individual confounders (18). The World Meteorological Organization (WMO) and World Health Organization (WHO) jointly recommend establishing graded early warning systems based on exposure-response relationships as a core strategy for addressing extreme weather health risks (19). Given the significant regional heterogeneity in the temperature-CO poisoning relationship across China, there is an urgent need for region-specific warning thresholds (8). Recent studies have highlighted the importance of understanding temperature-health relationships across diverse populations (20–22), underscoring the need for region-specific analyses such as the present study.

Despite this established knowledge, current CO poisoning prevention relies primarily on *post-hoc* case investigations or indoor CO alarm installations—approaches that are reactive or limited in coverage. Whether routinely monitored outdoor ambient temperature can serve as a low-cost, population-level proxy indicator for conditions associated with elevated CO poisoning risk remains unexplored in the Sichuan Basin. If validated, such a proxy would enable meteorological data—already collected in real time by weather services—to inform anticipatory public health messaging without requiring in-home monitoring infrastructure. The present study therefore examines whether outdoor temperature, as a readily measurable surrogate for cold-driven behavioral adaptations (heating device use and ventilation patterns), is associated with CO poisoning EMS responses at the population level, and whether exploratory temperature reference points can be derived to support local health meteorology services.

Using 13 years of CO poisoning EMS data (2013–2025) from Chengdu, this study employs a time-stratified case-crossover design combined with DLNM to quantify the temperature–CO poisoning association, identify susceptible subpopulations, estimate the attributable burden of temperatures deviating from the minimum morbidity temperature (hereafter termed ‘non-optimal' conditions), and derive exploratory temperature reference points for public health monitoring. This investigation directly responds to the WHO Climate Change and Health Action Framework and China's National Climate Change Health Adaptation Action Plan (2024–2030) (23), both of which prioritize localized early warning tools to reduce climate-sensitive health risks.

## 2 Materials and methods

### 2.1 Data sources

#### 2.1.1 CO poisoning emergency data

CO poisoning EMS records were obtained from the Chengdu First Aid Command Center from January 1, 2013 to December 31, 2025. Data covered all districts and counties of Chengdu and included: date of call, patient sex, age, address, chief complaint, and preliminary diagnosis. Inclusion criteria were chief complaint or preliminary diagnosis containing keywords: “carbon monoxide poisoning”, “CO poisoning”, “gas poisoning” (a common term for CO poisoning in China), or “charcoal heating poisoning”. Notably, among the 4,557 raw records, 4,530 (99.4%) were diagnosed as “gas poisoning”, 27 (0.6%) as “carbon monoxide poisoning”, and no cases of natural gas (methane) poisoning were identified; the term “gas poisoning” in the Chinese context specifically refers to carbon monoxide exposure from incomplete combustion of coal or gas fuels. Given the acute nature of CO poisoning, the EMS call date was considered the poisoning date, consistent with previous studies (6, 8). All personal identifiers were removed before analysis. A total of 4,557 raw CO poisoning records were obtained. After excluding 205 cases (4.5%) with missing data on any key exposure variable (age, temperature, humidity, PM_2_.5, wind speed, precipitation), the final analytic sample comprised 4,352 cases. No imputation was performed for missing values to preserve the integrity of exposure-response estimation under the case-crossover design.

#### 2.1.2 Meteorological and air pollution data

Daily meteorological data (January 1, 2013–December 31, 2025) were obtained from the Chengdu Meteorological Office, including: daily mean temperature (°C), daily mean relative humidity (%), daily mean atmospheric pressure (hPa), daily mean wind speed (m/s), daily precipitation (mm), and sunshine duration (h). Daily air pollutant concentrations were obtained from the Chengdu Municipal Bureau of Ecological Environment for the same period, including: fine particulate matter (PM_2_.5), inhalable particulate matter (PM10), sulfur dioxide (SO_2_), nitrogen dioxide (NO_2_), carbon monoxide (CO), and ozone (8-h maximum, O3-8h). All monitoring stations covered all administrative districts of Chengdu, and daily city-wide averages were calculated using arithmetic means. Missing data rates for all meteorological and pollutant variables were < 0.5%, providing complete data for case-crossover matching.

### 2.2 Statistical analysis

#### 2.2.1 Study design

We employed a time-stratified case-crossover design. This design uses each case as its own control by comparing exposure on the case day (poisoning date) with exposures on selected control days within the same time stratum. This approach effectively controls for all time-invariant individual confounders (e.g., age, sex, genetics, socioeconomic status) and eliminates confounding by season, long-term trends, and day-of-week effects (6, 18). For each CO poisoning case, the case day was defined as the poisoning date. Control days were selected from other dates within the same year, same month, and same day of the week. Each case and its 3–4 control days formed a stratum, which was included as a stratification variable in conditional logistic regression models.

#### 2.2.2 DLNM model specification

To simultaneously model the nonlinear effects of temperature and its lag structure, we applied a distributed lag nonlinear model (DLNM) (15, 16) using cross-basis functions to apply spline transformations to both the temperature and lag dimensions (24). The model was specified as follows (25):


$$\begin{array}{c} \text{logit}\left[\text{P}\left(\text{case }=\;1\right)\right]=\alpha +\text{cb}\left(\text{temp\_t},\text{lag}=5\right)+\text{ns}(\text{humidity},\\ \text{df}=3)+\text{ns}(\text{PM}2.5,\text{df}=3)+\text{wind speed}+\text{precipitation}\\ +\beta \text{DOW}+\gamma \text{Holiday}+\text{strata}(\text{stratum\_id}) \end{array}$$

Where *P* (case = 1) is the probability of being a case day within each matched stratum; α is the intercept; β and γ are coefficients for day of week and holiday indicators, respectively; cb (temp_t, lag = 5) is the cross-basis matrix for temperature and lag, with natural cubic splines for both the temperature dimension (df = 3) and lag dimension (df = 3), and maximum lag set to 5 days. The degrees of freedom for temperature and lag dimensions were selected based on the Akaike Information Criterion (AIC); df = 3 yielded the lowest AIC among tested values (2–4). The maximum lag of 5 days was chosen after comparing models with different lag lengths (3, 5, 7, 10 days). Although lag3 had a marginally lower AIC (Supplementary Table S4), the difference was small, and a longer lag window (5 days) was preferred to adequately capture the cumulative cold effect, consistent with previous studies (6, 7) and the observed single-day lag decay pattern. ns denotes natural cubic spline functions for humidity (df = 3) and PM_2_.5 (df = 3); wind speed and precipitation were included as linear terms to avoid overfitting given their relatively weak and monotonic associations with the outcome; DOW and Holiday were categorical variables; and strata (stratum_id) represents the fixed effect for each matching stratum, which is automatically controlled in conditional logistic regression (6, 18). PM_2_.5 was selected as the representative air pollutant due to its high correlation with other pollutants (PM10, NO_2_, SO_2_, CO) in Chengdu (r > 0.65; Supplementary Figure S2) and its established health relevance (10). A three-dimensional representation of the estimated temperature-lag-response surface is provided in Supplementary Figure S1. Sensitivity analyses confirmed that alternative lag specifications yielded directionally consistent estimates (Supplementary Table S4).

#### 2.2.3 Extreme temperature definition

Following previous studies (26–28), extreme cold was defined as the 2.5th percentile and extreme heat as the 97.5th percentile of the daily mean temperature distribution in the analysis dataset (case days and control days). This approach ensures consistency with the reference temperature (also derived from the analysis dataset) and reflects the exposure range actually used in the conditional logistic regression models. The median temperature of the analysis dataset (10.5 °C) was used as the reference temperature.

#### 2.2.4 Subgroup analyses

To identify susceptible populations, stratified analyses were conducted by age (< 20, 20–39, 40–59, ≥60 years), sex (male, female), season (spring: March–May; summer: June–August; autumn: September–November; winter: December–February), and humidity tertiles (low: < 66%; medium: 66%−77%; high: >77%), and wind speed (dichotomized at the median into low-wind and high-wind groups to explore potential ventilation-mediated effect modification). To avoid overfitting in subgroups with limited sample sizes, we implemented a sample size-adaptive modeling strategy, as recommended in recent environmental health studies (20). For subgroups with < 300 cases, maximum lag was set to 2 days with df = 2 for both temperature and lag dimensions, and prediction range restricted to the 10th−90th percentiles; for 300–500 cases, lag = 3 days, df = 2, and prediction range 5th−95th percentiles; for ≥500 cases, full parameters (lag = 5 days, df = 3) were applied. For comparability, all subgroups ultimately reported cumulative lag 0–5 day effects.

#### 2.2.5 Temperature-associated burden analysis

We calculated the attributable fraction (AF) and attributable number (AN) for different temperature ranges following standard methods (29). For a given temperature interval with cumulative relative risk RR = exp(∑β_x), AF = (RR – 1)/RR, and AN = *n* × AF, where n is the number of cases in that interval.

To estimate 95% CIs for AN, we used Monte Carlo simulations (30, 31). One thousand coefficient vectors were randomly sampled from the multivariate normal distribution *N*(β, Cov(β)) of the model coefficients. For each sample, AF and AN were recalculated for all temperature intervals, and the 2.5th and 97.5th percentiles of the 1,000 estimates were taken as the 95% CI.

The minimum morbidity temperature (MMT), defined as the temperature corresponding to the lowest point on the cumulative exposure-response curve, was estimated at 14.9 °C from the cumulative lag 0–5 days effect. Notably, the MMT is an empirically derived value and is distinct from the reference temperature (10.5 °C) used as the baseline for relative risk calculations. The MMT was used as the natural cutoff between cold and heat effects. Based on previous studies (13, 27, 28), we selected the 2.5th, 5th, 25th, 75th, 95th, and 97.5th percentiles of the daily temperature distribution as auxiliary cut points to ensure stable estimates across intervals. This resulted in eight temperature intervals:

Extreme cold ( ≤ 2.5th percentile).
Severe cold (>2.5th−5th percentile).
Moderate cold (>5th−25th percentile).
Mild cold (>25th percentile–MMT).
Mild heat (>MMT−75th percentile).
Moderate heat (>75th−95th percentile).
Severe heat (>95th−97.5th percentile).
Extreme heat (>97.5th percentile).

#### 2.2.6 Exploratory temperature reference point determination

Following the risk ratio increment method proposed by Lin et al. (32) and adapted by Chen et al. (33), we identified exploratory temperature reference points where the cumulative RR first exceeded 1.5-fold and 2.0-fold relative to the MMT. These exploratory reference points were defined as exploratory Tier-1 and Tier-2, respectively. Similar graded approaches based on exposure-response relationships have been applied in establishing meteorological health indices, where health risks are stratified by temperature percentiles (26). This approach aligns with WMO/WHO guidelines for graded early warning systems (19).

#### 2.2.7 Sensitivity analysis

To assess model robustness, we conducted systematic sensitivity analyses by: varying the degrees of freedom for humidity (2, 4); changing the maximum lag days (3, 7, 10) while keeping the degrees of freedom for temperature and lag dimensions fixed at 3 (the same as the main model) to isolate the effect of lag window length; and modifying degrees of freedom combinations for temperature and lag dimensions (temperature df = 4 with lag df = 3, and temperature df = 3 with lag df = 4) while keeping the maximum lag fixed at 5 days. Key subgroups were also tested with alternative parameter settings. All sensitivity analysis results are presented in Supplementary Tables S3 and S4, confirming the robustness of the main findings.

#### 2.2.8 Bootstrap sensitivity analysis for temperature thresholds

To evaluate the statistical robustness of the exploratory cold reference points (Tier-1: RR > 1.5; Tier-2: RR > 2.0), we conducted a case-level bootstrap with 1,000 resamples with replacement. For each resample, the full conditional logistic regression model with distributed lag non-linear terms was refitted, and reference point temperatures were re-derived using the same algorithm as the primary analysis. The 95% confidence intervals were calculated from the empirical distribution of 999 successful replicates (convergence failure rate: 0.1%).

#### 2.2.9 Statistical software

All analyses were performed using R version 4.5.3, with the dlnm (15, 16), survival, splines, ggplot2, and MASS packages. Case-crossover matching was implemented using dplyr and lubridate. Statistical significance was set at two-sided α = 0.05.

## 3 Results

### 3.1 Descriptive characteristics

**Figure 1:**
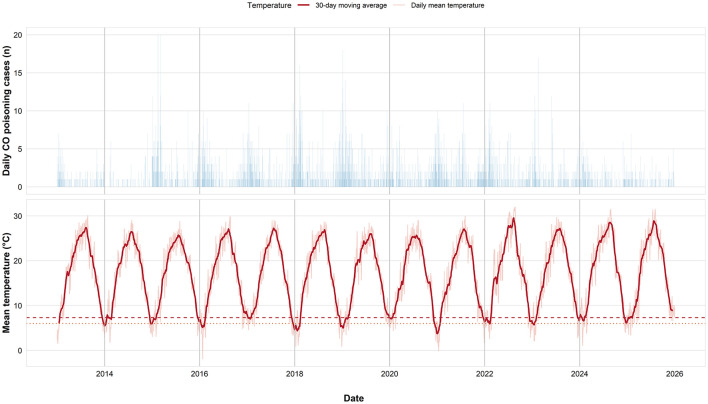
Time series of daily CO poisoning EMS responses (top panel) and daily mean temperature (bottom panel) in Chengdu, 2013–2025. In the top panel, daily case counts are shown as semi-transparent vertical bars. In the bottom panel, the light red line represents daily mean temperature and the thick dark red line shows the 30-day moving average, which serves as a population-level proxy for sustained cold exposure and associated behavioral adaptations (e.g., heating use, ventilation patterns). The red dashed line (7.3 °C) and orange dotted line (6.0 °C) indicate the exploratory Tier-1 and Tier-2 reference points derived from the exposure-response model. Bootstrap 95% confidence intervals for these reference points are reported in Supplementary Table S5 (Tier-1: 6.0–8.0 °C; Tier-2: 4.3–6.7 °C). Vertical gray lines mark even-numbered years for visual orientation.

After data cleaning, a total of 4,352 CO poisoning EMS cases were included in the final analysis, distributed across 1,894 days with an EMS response. The analysis dataset comprised 4,352 case-day records and 14,700 control-day records (total 19,052 records), with an average of 2.30 cases per day on days with at least one EMS response and 3.38 controls per case. Daily case numbers ranged from 1 to 20 (median: 2; IQR: 1–3). Winter (December–February) showed the highest case concentration (50.4%, Table 1). The time series of daily cases and temperature is shown in Figure 1.

**Table 1:** Baseline characteristics of CO poisoning EMS cases and environmental variables in Chengdu, 2013–2025.

Panel A. Case characteristics (*N* = 4,352)					
Characteristic		*n*		(%)	
Sex					
Male		2,072		(47.6)	
Female		2,280		(52.4)	
Age group (years)					
< 20		601		(13.8)	
20–39		2,221		(51.0)	
40–59		1,232		(28.3)	
≥60		298		(6.8)	
Season					
Spring		868		(20.0)	
Summer		690		(15.9)	
Autumn		600		(13.8)	
Winter		2,194		(50.4)	
Panel B. Meteorological and air pollutant variables (2013–2025; *N* = 4,748 calendar days).					
Variable	Min	Max	Median	IQR	Mean ±SD
Temperature (°C)	−1.9	32.0	17.6	10.2–23.5	17.0 ± 7.4
Relative humidity (%)	30.8	99.3	79.5	73.0–86.0	78.8 ± 9.7
PM_2_.5 (μg/m^3^)	3.5	381.0	38.5	23.8–63.2	50.4 ± 39.8
Wind speed (m/s)	0.2	4.4	1.2	1.0–1.5	1.3 ± 0.5
Precipitation (mm)	0.0	188.5	0.0	0.0–1.4	3.2 ± 11.1
Sunshine duration (h)	0.0	73.5	2.1	0.0–6.3	3.4 ± 3.8
PM10 (μg/m^3^)	5.5	726.0	62.7	40.2–97.1	76.2 ± 51.7
SO_2_ (μg/m^3^)	2.5	71.2	7.3	4.9–16.1	11.9 ± 10.1
NO_2_ (μg/m^3^)	6.9	91.1	32.6	23.5–42.6	34.1 ± 14.1
CO (mg/m^3^)	0.3	14.9	0.8	0.6–1.0	0.9 ± 0.5
O3-8 h (μg/m^3^)	10.1	278.0	83.8	57.4–127.6	94.0 ± 47.8
Panel C. Exposure distribution in the analysis dataset (19,052 records).					
Variable		Median	2.5th percentile	97.5th percentile	
Temperature (°C)		10.5	2.9	27.6	
Relative humidity (%)		79.5	59.4	94.8	
PM_2_.5 (μg/m^3^)		38.5	11.9	178.9	

IQR, interquartile range (25th−75th percentile); SD, standard deviation. The analysis dataset comprises 4,352 case-day records and 14,700 control-day records. Case characteristics are based on the analytic sample (*n* = 4,352). Meteorological and pollutant statistics in Panel B are based on all calendar days during the study period. Panel C presents the exposure distribution in the case-crossover analysis dataset; median values serve as reference points in the conditional logistic regression models.

Over the full study period (2013–2025), daily mean temperature ranged from −1.9 °C to 32.0 °C (median 17.6 °C, IQR 10.2–23.5 °C). Daily mean relative humidity ranged from 30.8% to 99.3% (median 79.5%, IQR 73.0–86.0%). Daily mean PM_2_.5 concentrations ranged from 3.5 to 381.0 μg/m^3^ (median 38.5 μg/m^3^, IQR 23.8–63.2 μg/m^3^), with pronounced seasonality: winter (December–February) median PM_2_.5 was 71.2 μg/m^3^ (IQR 46.7–104.9 μg/m^3^), while summer (June–August) median was 25.7 μg/m^3^ (IQR 18.2–37.1 μg/m^3^). Detailed statistics for all meteorological and pollutant variables are provided in Supplementary Table S1.

In the analysis dataset (case and control days), which determines the exposure distribution for the conditional logistic model, the median temperature was 10.5 °C (compared to 17.6 °C in the full study period). Extreme cold (2.5th percentile) was 2.9 °C, and extreme heat (97.5th percentile) was 27.6 °C. Baseline characteristics by sex, age, and season are presented in Table 1.

### 3.2 Correlation analysis

Pearson correlation analysis of all daily meteorological and pollutant data (2013–2025) revealed strong negative correlation between temperature and atmospheric pressure (r = −0.84) and strong positive correlation between temperature and O3 (r = 0.69). Humidity was negatively correlated with sunshine duration (r = −0.47). Air pollutants were moderately to highly correlated: PM_2_.5 correlated strongly with PM10 (r = 0.92), CO (r = 0.74), and NO_2_ (r = 0.77). Given that the model controlled for main confounders through spline functions and linear terms, and sensitivity analyses confirmed robustness, the final model included humidity, PM_2_.5, wind speed, and precipitation as covariates. The full correlation matrix among all meteorological and pollutant variables is displayed in Supplementary Figure S2.

### 3.3 Exposure-response relationship

**Figure 2:**
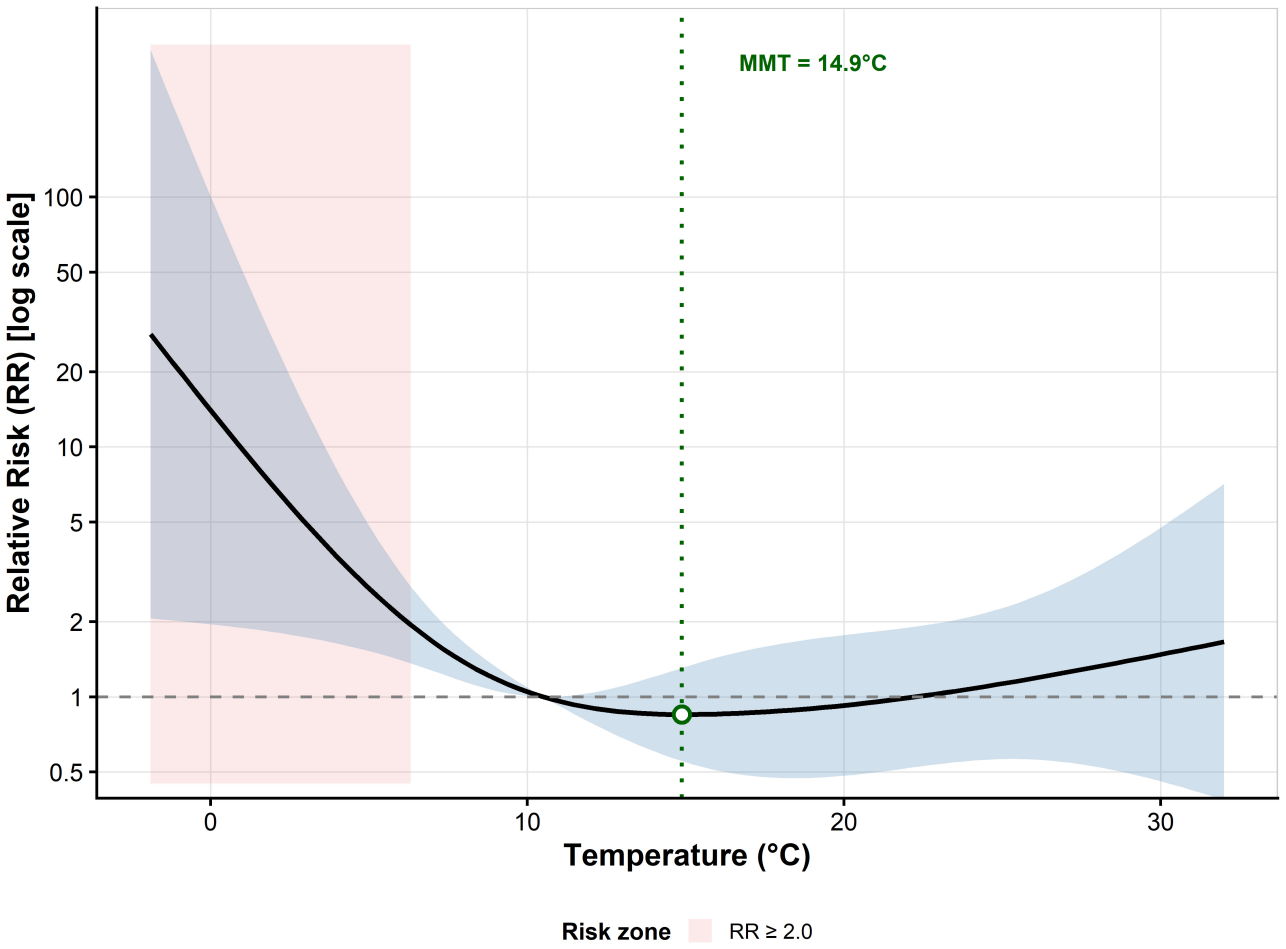
Exposure-response relationship between daily mean temperature and CO poisoning EMS responses (cumulative lag 0–5 days). The solid black line represents the estimated relative risk (RR), with the blue-shaded ribbon showing the 95% confidence interval. The dashed horizontal line indicates RR = 1 (reference). The vertical dotted line marks the minimum morbidity temperature (MMT = 14.9 °C). The light red shading highlights the temperature range where RR ≥2.0. Nominal exploratory reference points (Tier-1: 7.3 °C, RR = 1.55; Tier-2: 6.0 °C, RR = 2.11) and their bootstrap 95% confidence intervals (Tier-1: 6.0–8.0 °C; Tier-2: 4.3–6.7 °C; 1,000 resamples) are reported in Supplementary Table S5.

Using the analysis dataset median temperature (10.5 °C) as reference, the association between daily mean temperature and CO poisoning EMS responses exhibited a reverse “J” shape (Figure 2), with a minimum morbidity temperature (MMT) of 14.9 °C. Higher relative risks were observed at temperatures below 14.9 °C, while associations above 14.9 °C were modest with wider confidence intervals.

Relative risks at various temperature percentiles are shown in Table 2. CO poisoning risk increased with greater temperature deviation from the median, with RR = 7.22 (95%CI: 1.83–28.60) at the 1st percentile and RR = 1.39 (95%CI: 0.50–3.89) at the 99th percentile.

**Table 2:** Relative risks of CO poisoning EMS responses at different temperature percentiles (analysis dataset).

Percentile	Temperature (°C)	RR (95% CI)
1st	1.9	7.22 (1.83–28.60)
2.5th	2.9	5.12 (1.74–15.09)
5th	3.7	4.12 (1.68–10.13)
10th	4.9	2.76 (1.53–4.96)
50th (ref.)	10.5	1.00 (Reference)
90th	25.2	1.14 (0.56–2.30)
95th	26.6	1.22 (0.56–2.68)
97.5th	27.6	1.29 (0.54–3.10)
99th	28.9	1.39 (0.50–3.89)

RR, relative risk; CI, confidence interval. The RR estimates are derived from the cumulative lag 0–5 days exposure-response curve of the distributed lag nonlinear model. Reference temperature = 10.5 °C (median of the analysis dataset).

### 3.4 Single-day lag effects

Single-day lag effects for extreme cold (2.9 °C vs. reference 10.5 °C) showed that the peak effect occurred on the day of exposure (lag0), with RR = 1.79 (95%CI: 1.47–2.17). The effect decreased to RR = 1.46 at lag1 and approached 1 thereafter (Table 3, left columns). The complete single-day lag effects across all temperature percentiles, including 95% confidence intervals, are provided in Supplementary Table S2.

**Table 3:** Single-day and cumulative lag effects of extreme cold (2.9 °C vs. reference 10.5 °C) on CO poisoning EMS responses.

Lag day	Single-day RR (95% CI)	Cumulative lag window	Cumulative RR (95% CI)
0	1.79 (1.47–2.17)	lag0–1	2.62 (1.78–3.85)
1	1.46 (1.19–1.81)	lag0–2	3.27 (1.78–5.99)
2	1.25 (0.97–1.60)	lag0–3	3.76 (1.65–8.58)
3	1.15 (0.90–1.47)	lag0–4	4.31 (1.60–11.59)
4	1.15 (0.94–1.40)	lag0–5	5.12 (1.74–15.09)
5	1.19 (0.99–1.43)	—	—

RR, relative risk; CI, confidence interval. Extreme cold defined as 2.9 °C (2.5th percentile of the analysis dataset). Reference temperature = 10.5 °C (median of the analysis dataset). Estimates are derived from the distributed lag nonlinear model with natural cubic splines (temperature df = 3, lag df = 3, max lag = 5 days).

### 3.5 Cumulative lag effects

**Figure 3:**
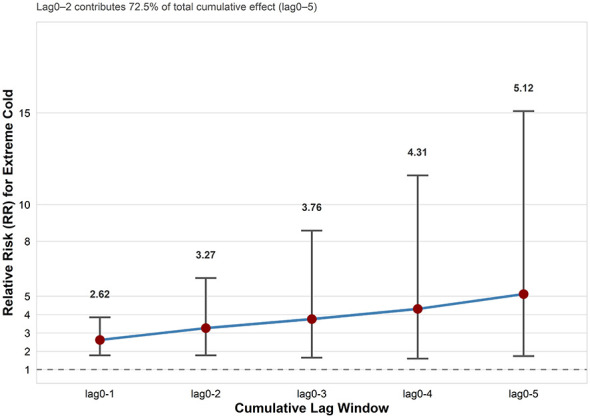
Cumulative relative risk (RR) of CO poisoning EMS responses for extreme cold exposure (2.9 °C, 2.5th percentile) compared with the minimum morbidity temperature (MMT = 14.9 °C), across cumulative lag windows. Error bars represent 95% confidence intervals. Numerical labels show point estimates of RR. The percentage contribution of lag0–2 to the total effect (lag0–5) was calculated as log(RR_lag0-2) / log(RR_lag0-5) × 100%, reflecting the additive decomposition of log-risks on the logarithmic scale.

The cumulative relative risk estimate for extreme cold was higher at longer lag windows (Table 3, right columns; Figure 3). The estimate increased from 2.62 (95% CI: 1.78–3.85) at lag0–1 to 5.12 (95% CI: 1.74–15.09) at lag0–5. The first 2 days (lag0–2, RR = 3.27) accounted for 72.5% of the total cumulative estimate (lag0–5), suggesting that the majority of the cold-related association was captured within 2 days post-exposure.

### 3.6 Subgroup analyses

All subgroup analyses were exploratory in nature due to reduced sample sizes within strata. Estimates with wide confidence intervals should be interpreted as hypothesis-generating rather than confirmatory evidence of effect modification.

#### 3.6.1 Age stratification

Age-stratified analyses showed significant cold effects in the 20–39 years group (RR = 3.76, 95%CI: 1.01–13.98) and ≥60 years group (RR = 1.63, 95%CI: 1.18–2.26). The 40–59 years group had the highest point estimate (RR = 6.19, 95%CI: 0.96–39.82), though with wide confidence intervals. The 40–59 years group also showed a significant heat effect (RR = 5.18, 95%CI: 1.15–23.39). Heat effects could not be estimated for the < 20 and ≥60 years groups due to sparse cases (Table 4).

**Table 4:** Age-stratified associations between extreme temperature and CO poisoning EMS responses (cumulative lag 0–5 days).

Age group	Cases	MMT (°C)	Cold effect RR (95% CI)	Heat effect RR (95% CI)
< 20	601	15.0	5.06 (0.39–65.36)	—^*^
20–39	2,221	27.5	3.76 (1.01–13.98)	0.87 (0.28–2.70)
40–59	1,232	10.4	6.19 (0.96–39.82)	5.18 (1.15–23.39)
≥60	298	19.6	1.63 (1.18–2.26)	—^*^

^*^MMT, minimum morbidity temperature; RR, relative risk; CI, confidence interval. “—” indicates that the heat effect could not be estimated due to sparse cases. MMT varies across subgroups because it is estimated as the minimum of each subgroup-specific exposure-response curve. Subgroup estimates, particularly those with wide confidence intervals, should be interpreted as exploratory and hypothesis-generating rather than definitive evidence of effect modification.

#### 3.6.2 Sex, season, and humidity stratification

**Figure 4:**
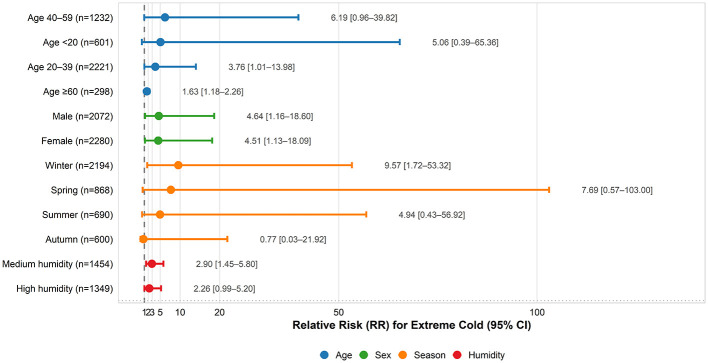
Forest plot of relative risks (RRs) for extreme cold exposure across demographic, seasonal, and humidity subgroups. Subgroups are ordered by descending RR magnitude within each category (Age, Sex, Season, Humidity). The dotted horizontal line separates primary demographic and seasonal findings from exploratory humidity stratifications. The dashed vertical line at RR = 1 indicates no association. Horizontal bars represent 95% confidence intervals. Numerical labels show point estimates and 95% CIs.

Stratified analyses by sex, season, and humidity revealed that cold effects were similar between females (RR = 4.51, 95% CI: 1.13–18.09) and males (RR = 4.64, 95% CI: 1.16–18.60), and heat effects did not reach statistical significance for either sex. Winter showed the strongest cold effect (RR = 9.57, 95% CI: 1.72–53.32), followed by spring (RR = 7.69, 95% CI: 0.57–103.00). Heat effects were most pronounced in autumn (RR = 5.18, 95% CI: 0.32–83.63) and summer (RR = 3.48, 95% CI: 0.30–40.38), though confidence intervals were wide. High humidity (>77%) significantly enhanced heat effects (RR = 3.15, 95% CI: 1.08–9.21) and showed borderline cold effects (RR = 2.26, 95% CI: 0.99–5.20). Medium humidity (66–77%) was associated with significant cold effects (RR = 2.90, 95% CI: 1.45–5.80). Low humidity (< 66%) showed no significant heat effect (RR = 0.81, 95% CI: 0.50–1.32).The extreme cold effects across all subgroups are summarized in Figure 4, and complete cold and heat effect estimates are provided in Supplementary Table S3.

#### 3.6.3 Wind speed stratification

Stratified analysis by wind speed revealed that the cumulative RR for extreme cold was higher on low-wind days (RR = 18.08, 95% CI: 2.69–121.65; *n* = 1,831) than on high-wind days (RR = 10.70, 95% CI: 1.42–80.48; *n* = 2,521). The minimum morbidity temperature (MMT) also differed between strata, being lower in the low-wind group (9.5 °C) compared with the high-wind group (14.1 °C). Formal interaction testing via likelihood ratio test did not reach statistical significance (χ^2^ = 3.6, df = 9, *p* = 0.94), likely reflecting limited power to detect multi-parameter interaction across the distributed lag structure given the rarity of extreme cold events. Nevertheless, the directional consistency—elevated risk and lower MMT under poor ventilation conditions—provides supplementary evidence supporting the hypothesis that ambient temperature may operate partly through a ventilation-mediated behavioral pathway for indoor CO accumulation. Complete wind-stratified estimates are provided in Supplementary Table S3.

### 3.7 Temperature-associated burden analysis

Temperature-associated burden analysis showed that non-optimal temperature was estimated to be associated with a substantial proportion of the CO poisoning burden in Chengdu (Table 5). The cold side (< 14.9 °C) was estimated to account for 1,178 cases (27.1% of total cases), while the heat side (>14.9 °C) accounted for 254 cases (5.8%). Overall, non-optimal temperature was estimated to be associated with 1,432 CO poisoning EMS responses, representing 32.9% of all cases (95% CI: 14.0%−50.5%). However, for temperature intervals from mild cold upward (mild cold, mild heat, moderate heat, severe heat, and extreme heat), the 95% confidence intervals for the attributable numbers included zero, indicating that these estimates are not statistically significant and should be interpreted with caution.

**Table 5:** Temperature-coincident cases by temperature interval, Chengdu 2013–2025.

Temperature interval	Center temperature (°C)^*^	Cases	Temperature-coincident cases, *n* (95% CI)
Extreme cold ( ≤ 2.5th)	2.0	123	108 (75–119)
Severe cold (2.5th−5th)	3.3	115	93 (63–106)
Moderate cold (5th−25th)	5.7	929	579 (299–736)
Mild cold (25th–MMT)	9.4	1,643	398 (0–896)
Mild heat (MMT−75th)	16.8	449	6 (0–64)
Moderate heat (75th−95th)	23.4	838	159 (0–411)
Severe heat (95th−97.5th)	27.0	130	42 (0–92)
Extreme heat (>97.5th)	28.6	125	47 (0–99)
Total	—^**^	4,352	1,432 (609–2,199)

^*^Center temperature is the mean temperature within each interval. Intervals are defined based on analysis dataset temperature percentiles. MMT, minimum morbidity temperature (14.9 °C); CI, confidence interval. ^**^“—” indicates not applicable. Temperature-coincident cases represent the estimated proportion of cases statistically associated with non-optimal temperature under the fitted model assumptions; these estimates should not be interpreted as the true causal burden given the absence of individual-level exposure and behavioral data. Confidence intervals that include zero indicate that the estimate is not statistically robust and should be interpreted with caution.

### 3.8 Exploratory reference points

Using the risk ratio increment method within the temperature range below the MMT (14.9 °C), we identified the points where RR first exceeded 1.5-fold and 2.0-fold. Based on the full-sample cumulative exposure-response curve, the nominal Tier-1 and Tier-2 cold reference points were identified at 7.3 °C (RR = 1.55, 95% CI: 1.23–1.96) and 6.0 °C (RR = 2.11, 95% CI: 1.41–3.16), respectively. Bootstrap validation (1,000 case-level resamples with replacement; convergence failure rate: 0.1%) yielded median estimates of 7.0 °C (95% CI: 6.0–8.0 °C) for the Tier-1 reference point and 6.0 °C (95% CI: 4.3–6.7 °C) for the Tier-2 reference point. The minor discrepancy between the nominal Tier-1 estimate (7.3 °C) and its bootstrap median (7.0 °C) reflects the inherent sampling variability at low temperatures, where extreme cold days are relatively sparse, and underscores the importance of interpreting these values as exploratory rather than precise operational thresholds. These reference points are illustrated in Figure 2. Although the two exploratory reference points differ by only 1.3 °C, this narrow interval corresponds to a sharp increase in risk from RR=1.55 to RR = 2.11, representing a 36% relative risk increment. Bootstrap sensitivity analysis confirmed the statistical robustness of both reference points across 1,000 case-level resamples (convergence failure rate: 0.1%). This nonlinear risk pattern supports the rationale for a two-tiered reference system: the exploratory Tier-1 point indicates a temperature at which cold-related risk begins to rise more steeply, at which point public reminders could be considered, while the exploratory Tier-2 point signals a doubling of risk, warranting coordinated multi-sectoral responses.

### 3.9 Sensitivity analysis

When the maximum lag days were varied (3, 7, 10) with degrees of freedom fixed at 3, the extreme cold cumulative RR estimates ranged from 2.95 to 3.58, which are lower than the main model estimate (5.12) but remain directionally consistent. The confidence intervals for longer lags (e.g., lag10: 0.48–26.59) overlapped with the main model's CI, supporting overall robustness despite attenuation of point estimates. Changing the degrees of freedom combinations (temperature df = 4, lag df = 3; temperature df = 3, lag df = 4) yielded RR estimates of 5.71 and 5.10, respectively. Varying humidity degrees of freedom (2, 4) and maximum lag days (3, 7, 10) resulted in extreme cold RR estimates ranging from 2.99 to 5.71, and attributable numbers ranging from 1,350 to 1,450, all close to the main model estimates, confirming model robustness. Longer lag periods (lag7, lag10) produced wider confidence intervals but point estimates remained directionally consistent with the main model. The attenuation of the effect estimate at lag10 (RR=3.58, 95% CI: 0.48–26.59) compared to the main model (RR = 5.12) suggests that the cold effect is primarily captured within 5 days, supporting our choice of lag = 5 days. Detailed results of sensitivity analyses for the main model and key subgroups are provided in Supplementary Table S4.

## 4 Discussion

This 13-year study of CO poisoning EMS responses in Chengdu provides a comprehensive characterization of the temperature-CO poisoning relationship, lag structure, population heterogeneity, and attributable burden in the Sichuan Basin. The main findings are: (1) The association between temperature and CO poisoning exhibited a reverse “J” shape with a minimum morbidity temperature of 14.9 °C, with low temperatures showing the strongest statistical association; (2) Cumulative lag analysis indicated that the majority of the cold-related association was captured within the first 2 days; (3) Subgroup analyses suggested potential vulnerability among young adults (20–39 years) and older adults (≥60 years), with winter and high-humidity conditions associated with higher risks; (4) Non-optimal temperatures were estimated to be statistically associated with 32.9% of all CO poisoning EMS responses; (5) Based on the exposure-response association, we propose exploratory cold reference points of 7.3 °C (bootstrap median: 7.0 °C) and 6.0 °C for Chengdu.

It is critical to emphasize that ambient temperature is not a direct biological cause of CO poisoning. Rather, it functions as a population-level proxy indicator for a constellation of unmeasured behavioral and environmental factors—including heating device usage, ventilation patterns, indoor-outdoor temperature differentials, and housing insulation quality—that collectively drive poisoning risk. The strong association observed in this study reflects the sensitivity of outdoor temperature as a surrogate for these proximal risk factors in the Sichuan Basin context, where decentralized heating and high winter humidity create conditions conducive to CO accumulation when temperatures drop. This interpretation aligns with established frameworks in environmental epidemiology that distinguish physiological from behavioral mediation pathways in temperature–health associations (34). Under this framework, the strong association observed between ambient temperature and CO poisoning is more plausibly attributed to cold-driven behavioral adaptations—such as increased use of unvented heating devices and reduced household ventilation—than to direct thermal physiology. However, we acknowledge that unmeasured time-varying confounders (e.g., daily fluctuations in heating intensity or ventilation habits) may partially drive this association, reinforcing the need to interpret these estimates as population-level risk markers rather than individual-level causal effects. A recent global systematic review further demonstrated that locally-defined extreme heat thresholds capture more context-specific risk patterns than fixed meteorological definitions (35), supporting the value of localized reference points in environmental health research broadly, including our application to CO poisoning in the Sichuan Basin. Consequently, the exploratory reference points and attributable fractions reported herein should be understood as statistical summaries of this proxy relationship, not as evidence of a direct etiological role of temperature.

### 4.1 Comparison with previous studies

The cumulative lag 0–5 day RR of 5.12 for extreme cold in our study is substantially higher than the pooled effect for southern regions (RR = 1.73) in the national 31-city study (8). This discrepancy may partly reflect regional climatic differences: Chengdu's high winter humidity, low sunshine duration, and lack of central heating may increase reliance on decentralized heating devices and improper usage (10). Compared to the Jinan study (cold spell OR = 2.53) (5), our higher effect estimate could partly be attributable to both the unique basin climate of Chengdu and methodological differences (continuous temperature analysis vs. event-based analysis). A recent retrospective analysis of CO poisoning in northern China similarly reported that low temperature was associated with increased risk, with each unit decrease in mean temperature corresponding to a 10.7% increase in poisoning risk (OR = 0.893, 95% CI: 0.873–0.914, *P* < 0.001), and 24-hour temperature fluctuations further associated with a further increase in risk of 25.7% per unit change (36). The Guangdong study reported lag effects persisting up to 7 days (RR range 2.24–3.81) (7), whereas our lag pattern was shorter (peak at lag0), possibly reflecting differences in heating practices between regions. Internationally, extreme cold spells have similarly driven CO poisoning surges through behavioral adaptations such as improper heater use and inadequate ventilation (37), supporting the generalizability of our findings.

### 4.2 Cumulative lag effects and acute nature

Cumulative lag analysis revealed the temporal accumulation of cold risk. The increase from RR = 2.62 at lag0–1 to 5.12 at lag0–5 demonstrates that cold effects accumulate over 5 days, but the first 2 days (lag0–2, RR = 3.27) already accounted for 72.5% of the total effect. This finding is consistent with previous research reporting associations between acute temperature extremes and increased emergency demand within hours (4) and has important implications for early warning timing.

### 4.3 Age and sex differences

The significant cold effects observed in young adults (20–39 years) and older adults (≥60 years) are generally consistent with Sichuan CO poisoning mortality data showing that adults aged 18–60 years account for 80.2% of deaths (14). Gansu province research similarly reported that extreme cold was associated with higher risks among adults ≥65 years and CO poisoning hospitalizations (13). The 40–59 years group had the highest point estimate (RR = 6.19) with wide confidence intervals, suggesting substantial variability within this group. A multi-community analysis in Guangdong also reported that middle-aged adults were particularly susceptible to temperature-related health burdens (38). Notably, occupational poisoning data from Sichuan indicate that the 41–50 years age group accounts for 40.55% of cases, with Chengdu ranking first among all prefectures (39), supporting the identification of middle-aged adults as a high-risk population in both occupational and non-occupational settings. No significant sex differences were observed. Given the wide confidence intervals for several age-specific estimates, these subgroup patterns should be interpreted as suggestive rather than definitive evidence of differential susceptibility.

### 4.4 Season, humidity, and wind speed interactions

The strong cold effect in winter (RR = 9.57) could be related to increased heating device usage (14). The significant heat effect under high humidity conditions (RR = 3.15) and significant cold effect under medium humidity (RR = 2.90) suggest important temperature-humidity interactions. Potential mechanisms include: (1) stagnant atmospheric conditions with high humidity favoring accumulation of indoor and outdoor pollutants (10); (2) humidity affecting combustion efficiency and CO production, and reducing ventilation frequency in heated indoor environments (40); (3) humidity-modified perceived temperature influencing heating or cooling behaviors; (4) during hot and humid weather in Chengdu, closed windows with air conditioning use may prolonge indoor CO accumulation from improperly installed gas water heaters, potentially explaining the observed heat-related risk. Ma et al. (36) found that temperature variability (24-h temperature change) was an independent risk factor for CO poisoning, while humidity alone showed no significant direct association (partial correlation −0.022, *P* = 0.464), suggesting that humidity effects may operate through interaction with temperature (36). A recent Taiwan study also reported that elevated temperatures significantly increased CO poisoning risk, though humidity was not independently associated (41). Our finding of enhanced heat effects under high humidity in a subtropical basin climate warrants validation in other regions. Additionally, wind speed stratification revealed a 69% higher extreme cold RR and a 4.6 °C lower MMT on low-wind days, consistent with reduced ventilation amplifying indoor CO accumulation. Although formal interaction testing was non-significant (*p* = 0.94), this likely reflects the low statistical power inherent to multi-parameter DLNM interaction terms when applied to sparse extreme-event data (16), rather than evidence of absent effect modification. In environmental epidemiology, directional consistency of stratified estimates is often considered more informative than formal *p*-values for hypothesis generation regarding mechanistic pathways (42). This directional alignment with the ventilation-behavior hypothesis reinforces the interpretation of ambient temperature as a proxy for household ventilation patterns rather than a direct physiological risk factor. Notably, low wind speed has been identified as a key predictor in operational heatwave forecasting models (43), lending external methodological support to our use of wind speed as an analogous proxy for ventilation-mediated CO poisoning risk in this context.

### 4.5 Public health implications: from reference points to action

Using the risk ratio increment method (32, 33), we identified nominal exploratory reference points at 7.3 °C (RR > 1.5) and 6.0 °C (RR > 2.0) for Chengdu. Bootstrap resampling yielded median estimates of 7.0 °C (95% CI: 6.0–8.0 °C) and 6.0 °C (95% CI: 4.3–6.7 °C), respectively (Supplementary Table S5; Supplementary Figure S3). The steep gradient between these two points supports a tiered rather than single-level approach, though prospective validation is necessary before operational implementation. These reference points align with China's National Climate Change Health Adaptation Action Plan (2024–2030) (23), which calls for strengthening early warning of climate-sensitive diseases through extreme weather forecasting. At the municipal level, they can be directly integrated into the “Health Meteorology·Co-construction Service” Cooperation Framework Agreement signed by the Health Commission of Chengdu and the Chengdu Meteorological Office in May 2025 (44), which establishes a joint disease forecasting system with provisions for issuing risk alerts when exploratory thresholds are reached.

Building on these policy frameworks, the proposed reference points may inform preliminary graded responses:

Tier-1 level ( ≤ 7.3 °C forecast for two consecutive days): Inform considerations for automated voice reminders to households using gas water heaters via community health hotlines or WeChat mini-programs, emphasizing ventilation and safe heating practices. Community health workers could intensify educational outreach in high-risk neighborhoods.

Tier-2 level ( ≤ 6.0 °C forecast): May warrant evaluation for coordinated multi-sectoral responses, including home visits by community health workers to inspect heating equipment in households with older adult members, pre-positioning of emergency medical resources, and public service announcements through local media.

These tiered actions align with the “risk-based graded response” principle and have been preliminarily incorporated into Chengdu's pilot health-meteorology platform (44). Pilot implementation could generate real-world data to validate the exploratory reference points' effectiveness, creating a feedback loop for iterative refinement.

### 4.6 Threshold uncertainty, implementation, and transferability

The exploratory reference points proposed here (7.3 °C and 6.0 °C) should be interpreted with several caveats. First, these values are derived from a single urban center in the Sichuan Basin and may not generalize to regions with different climate regimes, housing characteristics, or heating practices. The bootstrap 95% CIs (Tier-1: 6.0–8.0 °C; Tier-2: 4.3–6.7 °C) quantify sampling variability but do not capture uncertainties arising from unmeasured behavioral or structural factors. Second, prospective validation in independent cohorts and across multiple cities—particularly those within the same climatic zone (e.g., Chongqing, Guiyang)—is essential before operational deployment. Third, the threshold identification approach used here (risk ratio increment method) represents one of several possible strategies; alternative methods (e.g., receiver operating characteristic analysis, percentile-based cutoffs) may yield different reference values and warrant comparison in future work. Fourth, the integration of these reference points into existing health-meteorology platforms, such as Chengdu's pilot system, should be accompanied by real-world evaluation of their predictive performance, including sensitivity and specificity for CO poisoning events. Recent advances in operational climate-driven warning systems (43) and evidence supporting locally-defined thresholds over fixed meteorological definitions (35) provide a methodological foundation for such evaluation. Future prospective evaluation should assess the system's sensitivity, specificity, positive predictive value, and timeliness in detecting CO poisoning events when meteorological forecasts exceed the proposed reference points.

### 4.7 Global relevance and transferability

Chengdu's humid basin climate shares key characteristics with other regions worldwide, including the Yangtze River Delta, the Po Valley in Italy, and parts of Southeast Asia, where high winter humidity and low wind speeds combine with decentralized heating practices (10, 32). The reverse “J”-shaped exposure-response relationship and the pronounced cold effects observed in this study may therefore have transferable implications beyond Southwest China.

Recent studies have highlighted the importance of region-specific climate-health risk assessments across diverse geographic settings (20, 22). The methodological framework—time-stratified case-crossover design combined with DLNM—and the two-tiered exploratory reference point identification approach (7.3 °C and 6.0 °C) can be adapted to other regions with similar climatic and socioeconomic profiles. To facilitate such adaptation, we provide the R code used for exploratory reference point determination as Supplementary Code, allowing researchers to replicate the analysis with local data.

### 4.8 Strengths and limitations

Key strengths of this study include: (1) a large sample size with 13 years of data, providing stable estimates; (2) the time-stratified case-crossover design that effectively controls for time-invariant individual confounders (6, 18); (3) application of advanced DLNM methodology (15, 16); (4) rigorous subgroup analysis with sample size-adaptive modeling to avoid overfitting (20); (5) comprehensive attributable risk assessment using Monte Carlo simulations (30, 31); (6) detailed cumulative lag analysis; and (7) extensive sensitivity analyses confirming model robustness. The DLNM approach has been successfully applied to other environmental health questions in Southwest China, including a recent study examining the combined effects of temperature-humidity index and air quality on respiratory disease mortality in the subtropical monsoon climate of the southwest basin, further demonstrating the suitability and versatility of this methodology in our study region (45); (8) bootstrap validation confirming reproducibility of derived reference points (see Results 3.8). Furthermore, the case definition captured all CO exposure pathways regardless of fuel source; the observed temperature association therefore reflects the aggregate probability of heating-related behaviors rather than fuel-specific mechanisms.

Several limitations should be considered. First, exposure assessment and unmeasured factors. We used monitoring station data rather than individual-level measurements (ecological fallacy), and PM_2_.5 as a proxy for ambient CO due to the lack of direct CO concentration data, which may introduce residual confounding. Additionally, we lacked data on indoor temperature, heating behaviors, poisoning scenarios (e.g., residential vs. occupational), and could not distinguish between accidental and intentional (self-harm) CO poisoning, as EMS records do not capture intent. Time-varying practices such as daily ventilation or heating device usage on the exposure day were also not recorded (6, 18). Second, temporal resolution. Using EMS call date as the exposure date may misclassify cases with delayed care-seeking, though the acute nature of CO poisoning minimizes this bias. The absence of hourly data precluded analysis of diurnal effects. Third, statistical uncertainty in subgroups and attributable estimates. Sample size limitations in certain subgroups (≥60 years: *n* = 298; < 20 years heat effects: sparse cases) resulted in imprecise estimates. Moreover, the attributable fraction calculations rely on the counterfactual assumption of no unmeasured confounding, which cannot be satisfied in this observational design given the absence of indoor environmental and behavioral data. Therefore, AF and AN estimates represent the proportion of cases statistically attributable to non-optimal temperature under model assumptions, not the true causal burden. For the mild cold interval and all heat intervals, the 95% confidence intervals for attributable numbers included zero, further indicating that these estimates are not statistically robust and should be interpreted as hypothesis-generating rather than definitive burden assessments. Fourth, generalizability. As a single-city study, findings may not be directly transferable to other Sichuan Basin cities or regions with different climatic and socioeconomic profiles.

### 4.9 Future directions

Several avenues warrant further investigation. First, the proposed exploratory reference points (7.3 °C and 6.0 °C) should be validated in prospective studies that link real-time meteorological forecasts with EMS dispatch data to assess their predictive performance. Second, the modifying effects of indoor environmental factors—such as heating type, ventilation, and indoor-outdoor temperature differences—on CO poisoning risk remain largely unexplored. The recently launched Southwest China Indoor Environment Health Monitoring Project (2026–2030) offers an opportunity to address these gaps by collecting high-resolution indoor exposure data. Third, comparative studies across different climatic zones within China could help develop a nationally standardized but locally adaptive early warning system for CO poisoning. Finally, cost-effectiveness analyses are needed to evaluate the public health and economic benefits of implementing the proposed two-tiered warning system in Chengdu and other similar cities.

## 5 Conclusion

This study demonstrates a significant nonlinear association between daily mean temperature and CO poisoning EMS responses in Chengdu, with cold showing the strongest association. Risks are highest during winter and under high-humidity conditions. Young adults (20–39 years), middle-aged adults (40–59 years), and older adults (≥60 years) may benefit from targeted protective measures. Based on the exposure-response relationship and following international early warning guidelines, we propose nominal exploratory cold reference points of 7.3 °C (bootstrap median: 7.0 °C) and 6.0 °C to inform winter heating safety interventions in Chengdu. These exploratory reference points respond to the national call for localized CO poisoning temperature warning standards (8) and align with WMO/WHO recommendations for graded early warning systems (19). Future efforts could integrate these exploratory reference points with emergency medical resource allocation and public health education, leveraging ongoing national initiatives, such as the Indoor Environmental Health Impact Investigation and Protective Measure Effectiveness Assessment Project, which has been expanded nationwide under the Healthy Environment Promotion Action Implementation Plan (2025–2030) (46).

## Data Availability

The raw data supporting the conclusions of this article will be made available by the authors, without undue reservation.
