# Supplementary material for: Ambient temperature as a proxy indicator for carbon monoxide poisoning risk: deriving exploratory reference points for public health monitoring in Chengdu, China
Source: Front Public Health. 2026 Jul 15;14:1858128. doi: 10.3389/fpubh.2026.1858128 (PMC13415786; doi:10.3389/fpubh.2026.1858128)
Supplement: Supplementary file 1 [file Table_1.docx]

**Supplementary Material**

**1 Supplementary Tables**

**SUPPLEMENTARY TABLE S1.** Distribution of meteorological and air pollutant variables in the analysis dataset (case and control days, N = 19,052), Chengdu, 2013–2025.

| **Variable** | **Min** | **Max** | **Median** | **Q1** | **Q3** | **Mean** | **SD** | **2.5th** | **97.5th** |
| --- | --- | --- | --- | --- | --- | --- | --- | --- | --- |
| Temperature (°C) | –1.9 | 32.0 | 10.5 | 7.0 | 19.6 | 12.8 | 7.8 | 2.9 | 27.6 |
| humidity (%) | 30.8 | 99.3 | 79.5 | 73.0 | 86.0 | 78.8 | 9.7 | 59.4 | 94.8 |
| PM₂.₅ (μg/m³) | 3.5 | 381.0 | 38.5 | 23.8 | 63.2 | 50.4 | 39.8 | 11.9 | 178.9 |
| Wind speed (m/s) | 0.2 | 4.4 | 1.2 | 1.0 | 1.5 | 1.3 | 0.5 | 0.7 | 2.5 |
| Precipitation (mm) | 0.0 | 188.5 | 0.0 | 0.0 | 1.4 | 3.2 | 11.1 | 0.0 | 18.0 |

Note: This table describes the exposure distribution used in the conditional logistic regression models, which differs from the full study period distribution shown in main text Table 1 Panel B. Q1 = 25th percentile; Q3 = 75th percentile; SD = standard deviation. The 2.5th and 97.5th percentiles of temperature define extreme cold and extreme heat thresholds in the DLNM model.

**SUPPLEMENTARY TABLE S2.** Single-day lag effects at different temperature percentiles (RR

and 95% CI).

| **Percentile** | **Temp (°C)** | **Lag 0** | **Lag 1** | **Lag 2** | **Lag 3** | **Lag 4** | **Lag 5** |
| --- | --- | --- | --- | --- | --- | --- | --- |
| P1 | 1.9 | 1.97 (1.55–2.50) | 1.59 (1.22–2.07) | 1.33 (0.97–1.82) | 1.20 (0.88–1.65) | 1.18 (0.92–1.52) | 1.21 (0.97–1.52) |
| P2.5 | 2.9 | 1.79 (1.47–2.17) | 1.46 (1.19–1.81) | 1.25 (0.97–1.60) | 1.15 (0.90–1.47) | 1.15 (0.94–1.40) | 1.19 (0.99–1.43) |
| P5 | 3.7 | 1.66 (1.41–1.94) | 1.38 (1.16–1.63) | 1.19 (0.98–1.46) | 1.11 (0.91–1.36) | 1.12 (0.95–1.32) | 1.17 (1.01–1.36) |
| P10 | 4.9 | 1.48 (1.32–1.67) | 1.27 (1.13–1.43) | 1.12 (0.98–1.29) | 1.07 (0.93–1.22) | 1.09 (0.97–1.22) | 1.15 (1.03–1.28) |
| P25 | 7 | 1.25 (1.17–1.33) | 1.12 (1.06–1.19) | 1.04 (0.98–1.10) | 1.01 (0.95–1.07) | 1.04 (0.98–1.10) | 1.10 (1.03–1.17) |
| P50 (ref.) | 10.5 | 1.00 (—) | 1.00 (—) | 1.00 (—) | 1.00 (—) | 1.00 (—) | 1.00 (—) |
| P75 | 19.6 | 0.86 (0.72–1.03) | 0.97 (0.85–1.11) | 1.06 (0.92–1.22) | 1.08 (0.93–1.25) | 1.02 (0.89–1.17) | 0.93 (0.80–1.09) |
| P90 | 25.2 | 1.01 (0.79–1.30) | 0.99 (0.85–1.15) | 0.98 (0.85–1.13) | 1.00 (0.85–1.17) | 1.05 (0.89–1.23) | 1.12 (0.91–1.39) |
| P95 | 26.6 | 1.07 (0.81–1.42) | 0.99 (0.83–1.17) | 0.94 (0.80–1.11) | 0.97 (0.81–1.15) | 1.06 (0.88–1.27) | 1.20 (0.94–1.52) |
| P97.5 | 27.6 | 1.12 (0.82–1.53) | 0.99 (0.82–1.20) | 0.92 (0.77–1.10) | 0.94 (0.77–1.15) | 1.06 (0.87–1.30) | 1.26 (0.97–1.64) |
| P99 | 28.9 | 1.19 (0.84–1.69) | 1.00 (0.80–1.24) | 0.89 (0.72–1.10) | 0.91 (0.72–1.15) | 1.07 (0.85–1.35) | 1.35 (1.00–1.82) |

Note: RR = relative risk; CI = confidence interval. Reference temperature = 10.5°C (median of analysis dataset; P50). RR = 1.00 at P50 by definition. Estimates derived from distributed lag nonlinear model with natural cubic splines (temperature df = 3, lag df = 3, max lag = 5 days). **Bold** values indicate statistical significance (p < 0.05). "—" indicates not applicable.

**SUPPLEMENTARY TABLE S3.** Subgroup analyses of extreme temperature effects (cold and heat) on CO poisoning EMS responses (cumulative lag 0–5 days).

| **Subgroup** | **Cases** | **MMT (°C)** | **Cold effect RR (95% CI)** | **Heat effect RR (95% CI)** |
| --- | --- | --- | --- | --- |
| **Sex** |  |  |  |  |
| Female | 2,280 | 14.4 | 4.51 (1.13–18.09) | 1.43 (0.48–4.29) |
| Male | 2,072 | 13 | 4.64 (1.16–18.60) | 1.11 (0.31–3.93) |
| **Season** |  |  |  |  |
| Spring | 868 | 14.8 | 7.69 (0.57–103.00) | 2.10 (0.21–21.39) |
| Summer | 690 | 25.6 | —* | 3.48 (0.30–40.38) |
| Autumn | 600 | 11.4 | 0.77 (0.03–21.92) | 5.18 (0.32–83.63) |
| Winter | 2,194 | 12 | 9.57 (1.72–53.32) | 0.53 (0.12–2.40) |
| **Humidity** |  |  |  |  |
| Low (<66%) | 1,549 | —* | —* | 0.81 (0.50–1.32) |
| Medium (66–77%) | 1,454 | 19.5 | 2.90 (1.45–5.80) | 0.60 (0.17–2.17) |
| High (>77%) | 1,349 | 11.8 | 2.26 (0.99–5.20) | 3.15 (1.08–9.21) |
| **Wind speed** |  |  |  |  |
| Low wind | 1,831 | 9.5 | 18.08 (2.69–121.65) | — |
| High wind | 2,521 | 14.1 | 10.70 (1.42–80.48) | — |

Note: MMT = minimum morbidity temperature; RR = relative risk; CI = confidence interval. "—" indicates effect could not be estimated due to sparse cases (<5 events in relevant temperature range) or no exposure variation. MMT varies across subgroups because it is estimated as the minimum of each subgroup-specific exposure-response curve. Subgroup-specific modeling parameters were adapted based on sample size per Methods Section 2.2.4: <300 cases (max lag = 2, df = 2); 300–500 cases (max lag = 3, df = 2); ≥500 cases (max lag = 5, df = 3). All estimates reported as cumulative lag 0–5 days for comparability. Formal interaction tests were not conducted for season and humidity strata due to limited power; stratified results are presented for hypothesis generation. Wind speed stratification was performed at the median; likelihood ratio test for temperature cross-basis × wind group interaction: χ² = 3.6, df = 9, p = 0.936. Subgroup estimates with wide CIs should be interpreted as exploratory and hypothesis-generating rather than definitive evidence of effect modification.

**SUPPLEMENTARY TABLE S4.**Sensitivity analyses of extreme cold cumulative lag 0–5 day RR under alternative model specifications and subgroup-specific settings.

**Panel A: Model parameter sensitivity**

| **Parameter setting** | **Extreme cold RR (95% CI)** |
| --- | --- |
| **Main model (lag5, df = 3/3)** | **5.12 (1.74–15.09)** |
| Varying maximum lag (df fixed at 3) |  |
| lag3 | 2.95 (1.52–5.75) |
| lag7 | 3.17 (0.76–13.25) |
| lag10 | 3.58 (0.48–26.59) |
| Varying degrees of freedom (max lag fixed at 5) |  |
| temp df = 4, lag df = 3 | 5.71 (1.95–16.69) |
| temp df = 3, lag df = 4 | 5.10 (1.74–14.97) |

**Panel B: Subgroup sensitivity (main model vs. conservative lag3 specification)**

| **Subgroup** | **Cases** | **Main model (lag5, df = 3/3) RR (95% CI)** | **Conservative (lag3, df = 3/3) RR (95% CI)** |
| --- | --- | --- | --- |
| **Age** |  |  |  |
| <20 | 601 | 5.54 (0.36–85.50) | 1.66 (0.30–9.06) |
| 20–39 | 2,221 | 3.82 (1.01–14.47) | 1.60 (0.66–3.88) |
| 40–59 | 1,232 | 6.27 (0.96–40.93) | 4.53 (1.42–14.40) |
| ≥60 | 298 | 8.41 (0.25–281.88) | 5.30 (0.50–55.70) |
| **Sex** |  |  |  |
| Female | 2,280 | 4.47 (1.13–17.74) | 2.84 (1.22–6.59) |
| Male | 2,072 | 4.58 (1.16–18.09) | 3.02 (1.22–7.51) |
| **Season** |  |  |  |
| Spring | 868 | 7.96 (0.57–110.60) | 1.39 (0.27–7.13) |
| Summer | 690 | 4.95 (0.43–57.11) | 1.06 (0.21–5.34) |
| Autumn | 600 | 0.78 (0.03–22.60) | 4.67 (0.82–26.52) |
| Winter | 2,194 | 9.73 (1.72–55.01) | 3.20 (1.25–8.17) |
| **Humidity** |  |  |  |
| Low | 1,432 | 3.87 (0.41–36.74) | 1.68 (0.33–8.68) |
| Medium | 1,515 | 17.13 (0.81–361.37) | 5.73 (0.73–44.89) |
| High | 1,405 | 1.16 (0.06–23.33) | 0.84 (0.11–6.35) |

Note: RR = relative risk; CI = confidence interval. **Bold** indicates the main model specification in Panel A. In Panel A, "Varying maximum lag" analyses used temperature and lag df fixed at 3. "Varying df" analyses used max lag fixed at 5 days. AIC values are available from the corresponding author upon request. In Panel B, the main model uses max lag = 5 days, temperature df = 3, lag df = 3. The conservative specification uses max lag = 3 days with the same df. Estimates with extremely wide CIs (e.g., ≥60 years, spring, medium humidity) reflect limited case counts in certain subgroups and should be interpreted as indicative of direction rather than precise magnitude. Full parameter sweep results (lag7, lag10, additional df variations) are available from the corresponding author upon request.

**SUPPLEMENTARY TABLE S5.**Bootstrap sensitivity analysis of exploratory temperature reference points (1,000 case-level resamples).

| **Reference point** | **Nominal (°C)** | **Bootstrap median (°C)** | **Bootstrap 95% CI (°C)** | **Convergence failure** |
| --- | --- | --- | --- | --- |
| Tier-1 (RR > 1.5) | 7.3 | 7 | 6.0–8.0 | 0.10% |
| Tier-2 (RR > 2.0) | 6 | 6 | 4.3–6.7 | 0.10% |

Note: CI = confidence interval; RR = relative risk. Bootstrap percentiles were calculated from 999 successful replicates (1/1,000 convergence failure). The nominal estimates are derived from the full-sample exposure-response curve, while the bootstrap medians and 95% CIs reflect the sampling variability of these exploratory reference points.

**2 Supplementary Figure Legends**

**Supplementary Figure S1.** Three-dimensional temperature-lag-response surface for the association between daily mean temperature and CO poisoning EMS responses. Relative risk (RR) is shown on the z-axis, with the semi-transparent gray plane at RR = 1 indicating no association. The color scale represents deviation from RR = 1: blue/purple = lower risk (RR < 1), yellow/red = higher risk (RR > 1). The surface is based on the distributed lag non-linear model (DLNM) with natural cubic splines (temperature df = 3, lag df = 3, max lag = 5 days).

**Supplementary Figure S2.** Correlation heatmap of meteorological and air pollutant variables in Chengdu, 2013–2025. Pearson correlation coefficients are shown in the lower triangle. The color scale ranges from blue (negative correlation) through white (no correlation) to red (positive correlation). Numerical labels display the exact correlation coefficients.

**Supplementary Figure S3.** Bootstrap distributions of the Tier-1 (RR > 1.5) and Tier-2 (RR > 2.0) exploratory temperature reference points based on 1,000 case-level resamples with replacement. Solid blue lines indicate point estimates (medians of bootstrap distributions); dotted gray lines mark 95% confidence interval boundaries. Numerical CI values and convergence failure rate are provided in the figure caption.

**3 R code used for determining exploratory temperature reference points**

# Load required packages

library(dlnm) # Distributed lag non-linear models

library(survival) # Conditional logistic regression (clogit)

library(splines) # Natural cubic splines

library(dplyr) # Data manipulation

# --------------------------------------------------------

# Step 1: Prepare the data

# --------------------------------------------------------

# The dataset 'df' should contain the following variables:

# - is_case: 1 for case day, 0 for control day

# - temp: daily mean temperature (°C)

# - humidity: daily mean relative humidity (%)

# - PM25: daily mean PM2.5 concentration (μg/m³)

# - wind: daily mean wind speed (m/s)

# - rain: daily precipitation (mm)

# - dow_name: day of week (factor)

# - holiday: holiday indicator (0/1)

# - stratum_id: matching stratum identifier

# Example: Load your matched case-crossover dataset

# df <- read.csv("matched_case_crossover_data.csv")

# --------------------------------------------------------

# Step 2: Build the cross-basis for temperature

# --------------------------------------------------------

# Set modeling parameters (as described in the manuscript)

max_lag <- 5 # Maximum lag days

df_var <- 3 # Degrees of freedom for temperature dimension

df_lag <- 3 # Degrees of freedom for lag dimension

# Create the cross-basis matrix

cb_temp <- crossbasis(

x = df$temp,

lag = max_lag,

argvar = list(fun = "ns", df = df_var),

arglag = list(fun = "ns", df = df_lag)

)

# --------------------------------------------------------

# Step 3: Fit the main conditional logistic regression model

# --------------------------------------------------------

model_main <- clogit(

is_case ~ cb_temp + ns(humidity, df = 3) + ns(PM25, df = 3) +

wind + rain + dow_name + holiday + strata(stratum_id),

data = df,

method = "efron"

)

# --------------------------------------------------------

# Step 4: Predict the cumulative exposure-response curve

# --------------------------------------------------------

# Define reference temperature (median of the analysis dataset)

ref_temp <- median(df$temp, na.rm = TRUE)

# Define temperature sequence for prediction

temp_seq <- seq(

min(df$temp, na.rm = TRUE),

max(df$temp, na.rm = TRUE),

length = 100

)

# Obtain cumulative predictions

pred_main <- crosspred(

basis = cb_temp,

model = model_main,

at = temp_seq,

cen = ref_temp,

cumul = TRUE

)

# Extract the overall cumulative RR and confidence intervals

plot_data <- data.frame(

temp = temp_seq,

RR = as.numeric(pred_main$allRRfit),

RR_low = as.numeric(pred_main$allRRlow),

RR_high = as.numeric(pred_main$allRRhigh)

)

# --------------------------------------------------------

# Step 5: Identify the minimum morbidity temperature (MMT)

# --------------------------------------------------------

mmt <- plot_data$temp[which.min(plot_data$RR)]

cat("Minimum Morbidity Temperature (MMT):", round(mmt, 1), "°C\n")

# --------------------------------------------------------

# Step 6: Determine exploratory reference points below MMT

# --------------------------------------------------------

# Subset data to temperatures below MMT, ordered from high to low

cold_side <- plot_data[plot_data$temp < mmt, ]

cold_side <- cold_side[order(cold_side$temp, decreasing = TRUE), ]

# Tier-1 reference point: RR first exceeds 1.5

primary_idx <- which(cold_side$RR > 1.5)[1]

if (!is.na(primary_idx)) {

primary_temp <- cold_side$temp[primary_idx]

primary_RR <- cold_side$RR[primary_idx]

primary_low <- cold_side$RR_low[primary_idx]

primary_high <- cold_side$RR_high[primary_idx]

cat("\n=== Tier-1 Exploratory Reference Point (RR > 1.5) ===\n")

cat("Temperature:", round(primary_temp, 1), "°C\n")

cat("RR:", round(primary_RR, 2),

"(95% CI:", round(primary_low, 2), "-", round(primary_high, 2), ")\n")

}

# Tier-2 reference point: RR first exceeds 2.0

advanced_idx <- which(cold_side$RR > 2.0)[1]

if (!is.na(advanced_idx)) {

advanced_temp <- cold_side$temp[advanced_idx]

advanced_RR <- cold_side$RR[advanced_idx]

advanced_low <- cold_side$RR_low[advanced_idx]

advanced_high <- cold_side$RR_high[advanced_idx]

cat("\n=== Tier-2 Exploratory Reference Point (RR > 2.0) ===\n")

cat("Temperature:", round(advanced_temp, 1), "°C\n")

cat("RR:", round(advanced_RR, 2),

"(95% CI:", round(advanced_low, 2), "-", round(advanced_high, 2), ")\n")

}

# --------------------------------------------------------

# Step 7 (Optional): Bootstrap sensitivity analysis

# --------------------------------------------------------

# This section provides a simplified example of the bootstrap

# procedure used to evaluate the robustness of the reference points.

# For the full implementation with 1,000 resamples, please refer to

# the complete analysis code available from the corresponding author.

# set.seed(123)

# n_boot <- 1000

# boot_primary <- numeric(n_boot)

# boot_advanced <- numeric(n_boot)

#

# case_ids <- unique(df$case_id[df$is_case == 1])

#

# for (i in 1:n_boot) {

# boot_ids <- sample(case_ids, size = length(case_ids), replace = TRUE)

# df_boot <- df %>% filter(case_id %in% boot_ids)

#

# # Re-fit model and re-derive thresholds

# # ... (same steps as above)

#

# boot_primary[i] <- primary_temp_boot

# boot_advanced[i] <- advanced_temp_boot

# }

#

# # Calculate 95% bootstrap confidence intervals

# primary_ci <- quantile(boot_primary, c(0.025, 0.975), na.rm = TRUE)

# advanced_ci <- quantile(boot_advanced, c(0.025, 0.975), na.rm = TRUE)

# cat("\n=== Bootstrap 95% Confidence Intervals ===\n")

# cat("Tier-1:", round(primary_ci[1], 1), "-", round(primary_ci[2], 1), "°C\n")

# cat("Tier-2:", round(advanced_ci[1], 1), "-", round(advanced_ci[2], 1), "°C\n")

# --------------------------------------------------------

# Step 8: Visualize the exposure-response curve with thresholds

# --------------------------------------------------------

library(ggplot2)

# Create risk zone background

rect_data <- data.frame(

xmin = c(min(plot_data$temp), advanced_temp),

xmax = c(advanced_temp, primary_temp),

risk = factor(c("RR ≥ 2.0", "1.5 ≤ RR < 2.0"))

)

# Plot

p <- ggplot() +

geom_rect(data = rect_data, aes(xmin = xmin, xmax = xmax,

ymin = 0.5, ymax = max(plot_data$RR_high) * 1.05,

fill = risk),

alpha = 0.10, color = NA) +

geom_ribbon(data = plot_data, aes(x = temp, ymin = RR_low, ymax = RR_high),

alpha = 0.25, fill = "steelblue") +

geom_line(data = plot_data, aes(x = temp, y = RR), color = "black", linewidth = 1) +

geom_hline(yintercept = 1, linetype = "dashed", color = "gray50", linewidth = 0.6) +

geom_vline(xintercept = mmt, linetype = "dotted", color = "darkgreen", linewidth = 0.8) +

geom_vline(xintercept = primary_temp, linetype = "dotted", color = "gray60", linewidth = 0.5) +

geom_vline(xintercept = advanced_temp, linetype = "dotted", color = "gray60", linewidth = 0.5) +

scale_fill_manual(name = "Risk zone",

values = c("RR ≥ 2.0" = "#D73027",

"1.5 ≤ RR < 2.0" = "#FC8D59"),

labels = c("RR ≥ 2.0", "1.5 ≤ RR < 2.0")) +

scale_y_continuous(trans = "log10",

breaks = c(0.5, 1, 2, 5, 10, 20, 50, 100),

name = "Relative Risk (RR) [log scale]") +

scale_x_continuous(name = "Temperature (°C)") +

annotate("text", x = mmt + 1.8, y = max(plot_data$RR_high) * 0.85,

label = paste0("MMT = ", round(mmt, 1), "°C"),

hjust = 0, size = 3.5, color = "darkgreen", fontface = "bold") +

annotate("text", x = primary_temp + 0.8, y = primary_RR * 1.5,

label = paste0("Tier-1: ", round(primary_temp, 1), "°C\nRR=", round(primary_RR, 2)),

hjust = 0, size = 3.5, color = "#FC8D59") +

annotate("text", x = advanced_temp + 0.8, y = advanced_RR * 1.5,

label = paste0("Tier-2: ", round(advanced_temp, 1), "°C\nRR=", round(advanced_RR, 2)),

hjust = 0, size = 3.5, color = "#D73027") +

theme_classic(base_size = 13) +

theme(legend.position = "bottom")

# Display the plot

print(p)

# Save the figure (optional)

# ggsave("Exposure_Response_Curve_with_Thresholds.png", p, width = 8, height = 6, dpi = 600)
