# Supplementary figures and images for: Ambient temperature as a proxy indicator for carbon monoxide poisoning risk: deriving exploratory reference points for public health monitoring in Chengdu, China
Source: Front Public Health. 2026 Jul 15;14:1858128. doi: 10.3389/fpubh.2026.1858128 (PMC13415786; doi:10.3389/fpubh.2026.1858128)

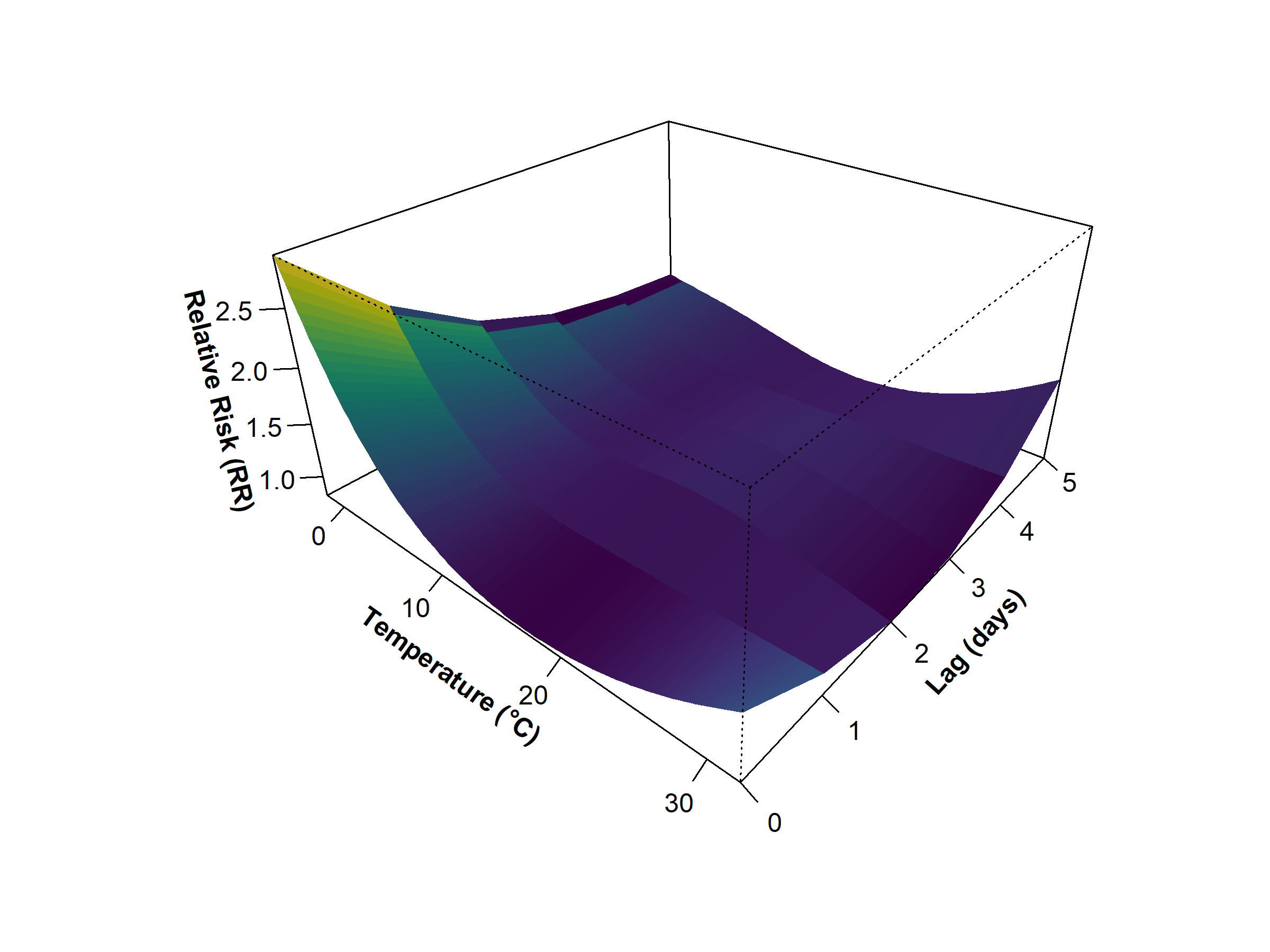

Supplement: Supplementary file 2 [file Image_1.tiff]

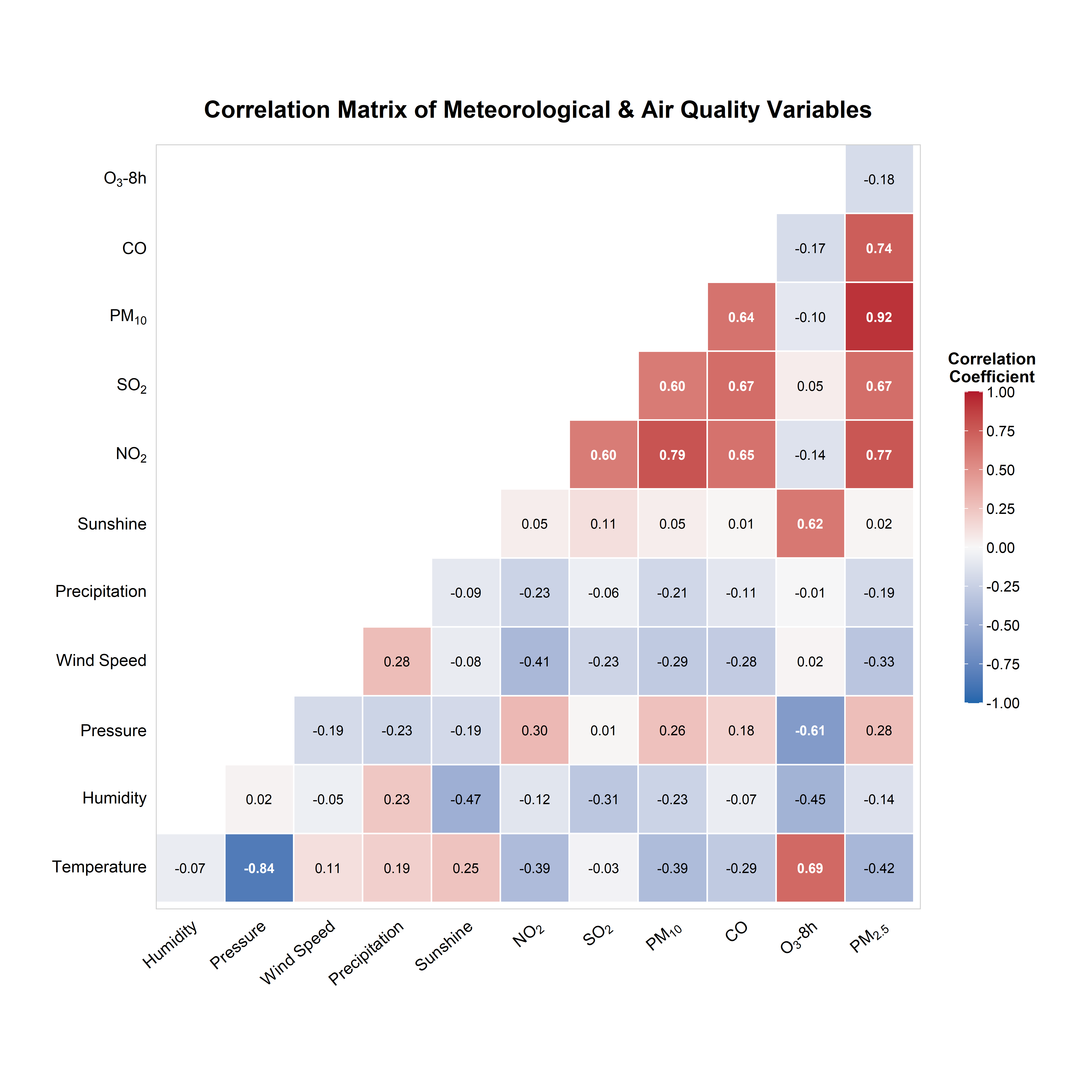

Supplement: Supplementary file 3 [file Image_2.tiff]

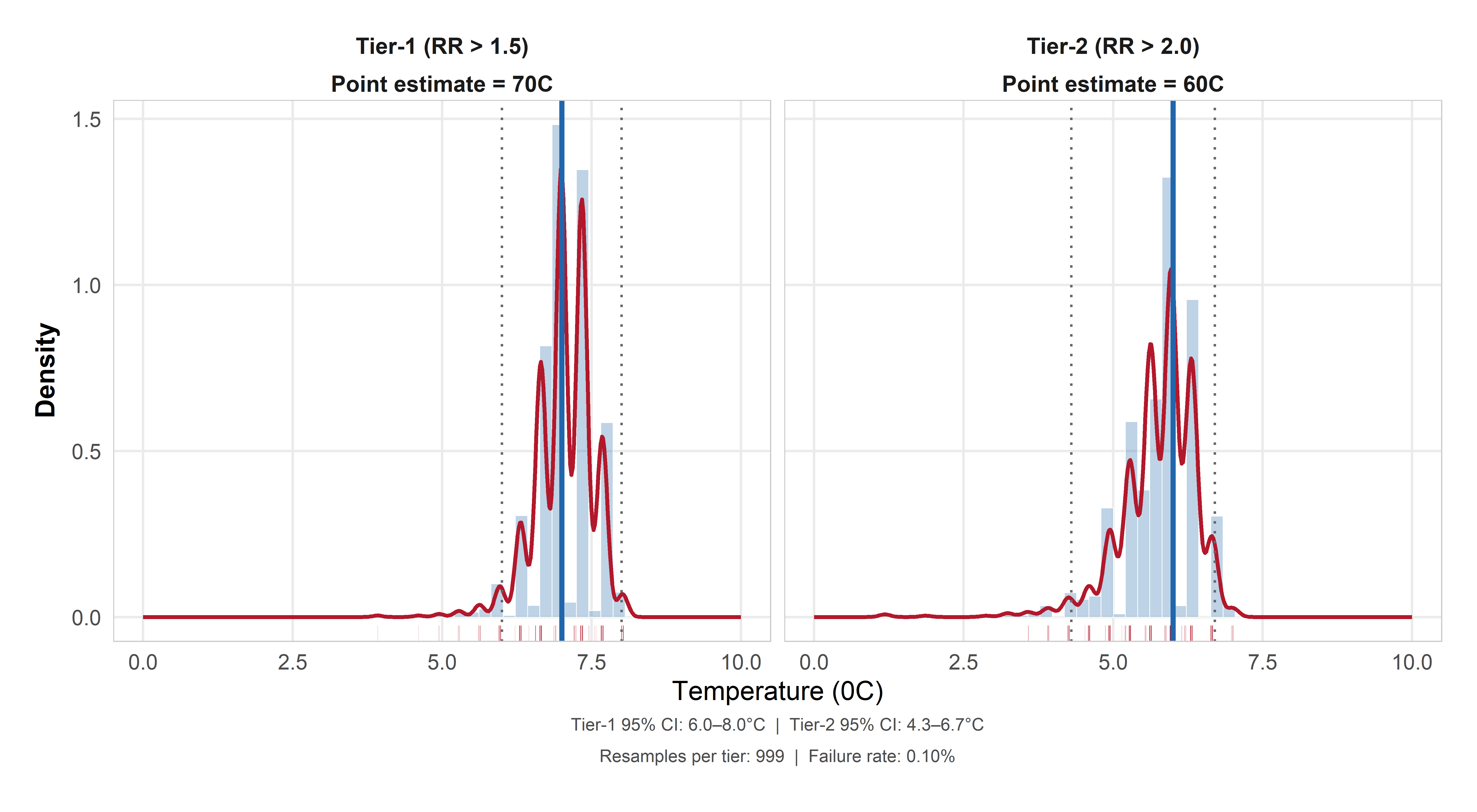

Supplement: Supplementary file 4 [file Image_3.tiff]
